# Supplementary material for: Splice-Junction-Based Mapping of Alternative Isoforms in the Human Proteome
Source: Cell Rep. Author manuscript; Available in PMC 2020 Jan 15. (PMC6961840; doi:10.1016/j.celrep.2019.11.026)
Supplement: 2 [file NIHMS1546469-supplement-2.zip › DF1/PXD009737/TestisGluC/TestisGluC_28_MUS81_PPPRNKRHCKRPLPACPNPLFVRWLTE.pdf]

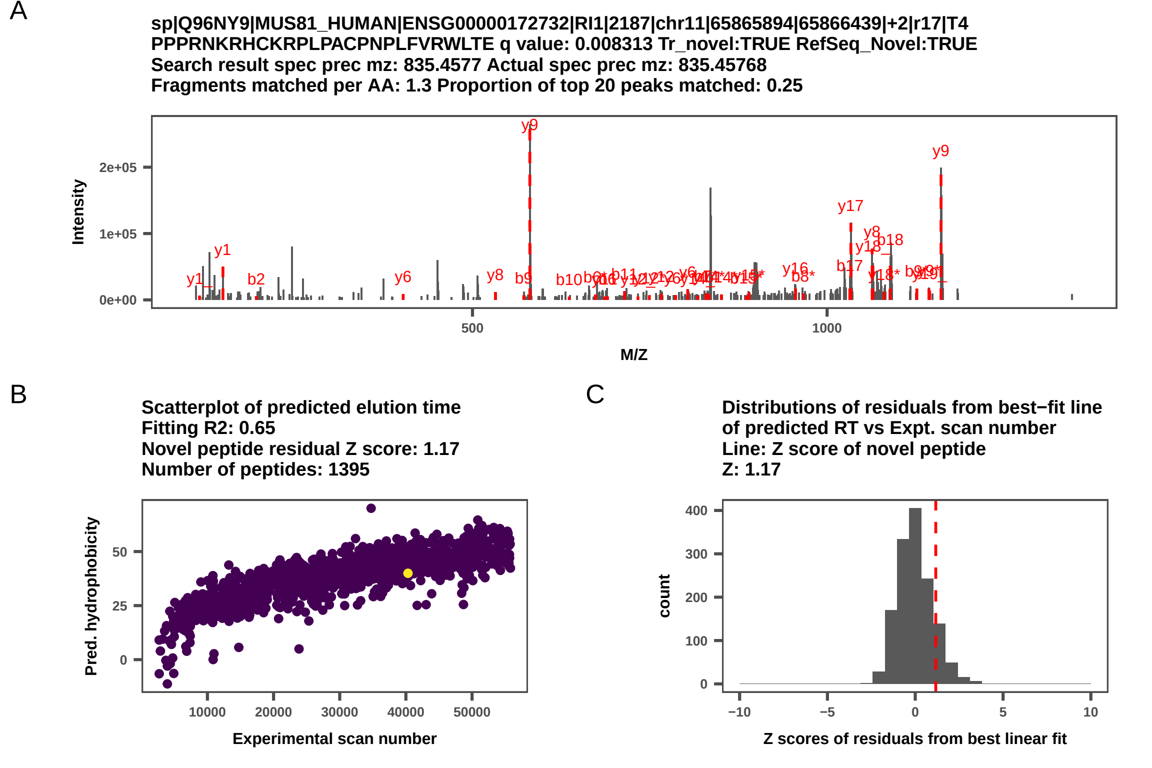

A
sp|Q96NY9|MUS81_HUMAN|ENSG00000172732|RI1|2187|chr11|65865894|65866439|+2|r17|T4
PPPRNKRHCKRPLPACPNPLFVRWLTE q value: 0.008313 Tr_novel:TRUE RefSeq_Novel:TRUE
Search result spec prec mz: 835.4577 Actual spec prec mz: 835.45768
Fragments matched per AA: 1.3 Proportion of top 20 peaks matched: 0.25
Intensity
2e+05
1e+05
0e+00
M/Z
500
1000
y9
y1
y2
b2
y6
y8
b9
b10
y17
y8
b18
b17
y16
b8*
B
Scatterplot of predicted elution time
Fitting R2: 0.65
Novel peptide residual Z score: 1.17
Number of peptides: 1395
Pred. hydrophobicity
50
25
0
10000
20000
30000
40000
50000
Experimental scan number
C
Distributions of residuals from best−fit line
of predicted RT vs Expt. scan number
Line: Z score of novel peptide
Z: 1.17
count
400
300
200
100
0
−10
−5
0
5
10
Z scores of residuals from best linear fit
